# Occurrence of antimicrobial resistance and antimicrobial resistance genes in methicillin-resistant *Staphylococcus aureus* isolated from healthy rabbits

**DOI:** 10.14202/vetworld.2022.2699-2704

**Published:** 2022-11-27

**Authors:** Arunee Jangsangthong, Nawarat Suriyakhun, Witawat Tunyong, Thida Kong-Ngoen, Sirijan Santajit, Nitaya Indrawattana, Shutipen Buranasinsup

**Affiliations:** 1 Department of Pre-clinic and Applied Animal Science, Faculty of Veterinary Science, Mahidol University, Nakornpathom 73710, Thailand; 2 Prasu-Arthorn Animal Hospital, Faculty of Veterinary Science, Mahidol University, Nakhonpathom 73170, Thailand; 3 Department of Microbiology and Immunology, Faculty of Tropical Medicine, Mahidol University, Bangkok 10400, Thailand; 4 Department of Medical Technology, School of Allied Health Sciences, Walailak University, Nakhon Si Thammarat 80160, Thailand; 5 Research Center in Tropical Pathobiology, Walailak University, Nakhon Si Thammarat 80160, Thailand

**Keywords:** antimicrobial resistance, antimicrobial-resistant genes, healthy rabbits, methicillin-resistant *Staphylococcus aureus*

## Abstract

**Background and Aim:**

Methicillin-resistant globally, *Staphylococcus aureus* (MRSA) is a major cause of disease in both humans and animals. Several studies have documented the presence of MRSA in healthy and infected animals. However, there is less information on MRSA occurrence in exotic pets, especially healthy rabbits. This study aimed to look into the antimicrobial resistance profile, hidden antimicrobial-resistant genes in isolated bacteria, and to estimate prevalence of MRSA in healthy rabbits.

**Materials and Methods:**

Two-hundreds and eighteen samples, including 42 eyes, 44 ears, 44 oral, 44 ventral thoracic, and 44 perineal swabs, were taken from 44 healthy rabbits that visited the Prasu-Arthorn Animal Hospital, in Nakornpathom, Thailand, from January 2015 to March 2016. The traditional methods of Gram stain, mannitol fermentation, hemolysis on blood agar, catalase test, and coagulase production were used to confirm the presence of *Staphylococcus aureus* in all specimens. All bacterial isolates were determined by antimicrobial susceptibility test by the disk diffusion method. The polymerase chain reaction was used to identify the antimicrobial-resistant genes (*bla*Z, *mec*A, *aac*A*-aph*D, *msr*A, *tet*K, *gyr*A, *grl*A, and *dfr*G) in isolates of MRSA with a cefoxitin-resistant phenotype.

**Results:**

From 218 specimens, 185 *S. aureus* were isolated, with the majority of these being found in the oral cavity (29.73%) and ventral thoracic area (22.7%), respectively. Forty-seven (25.41%) MRSAs were found in *S. aureus* isolates, with the majority of these being found in the perineum (16, 34.04%) and ventral thoracic area (13, 27.66%) specimens. Among MRSAs, 29 (61.7%) isolates were multidrug-resistant (MDR) strains. Most of MRSA isolates were resistant to penicillin (100%), followed by ceftriaxone (44.68%) and azithromycin (44.68%). In addition, these bacteria contained the most drug-resistance genes, *bla*Z (47.83%), followed by *gyr*A (36.17%) and *tet*K (23.4%).

**Conclusion:**

This study revealed that MRSA could be found even in healthy rabbits. Some MRSAs strains were MDR–MRSA, which means that when an infection occurs, the available antibiotics were not effective in treating it. To prevent the spread of MDR–MRSA from pets to owners, it may be helpful to educate owners about effective prevention and hygiene measures.

## Introduction

Early in the 1960s, methicillin-resistant *Staphylococcus aureus* (MRSA) was identified for the first time. It has become a serious nosocomial pathogen worldwide. The World Health Organization classified this pathogen as a serious pathogen that requires research and treatment. It is well-known that the *mec*A gene mediates the resistance of staphylococci to methicillin. This gene produces the penicillin-binding protein 2a, which has a low affinity for all-lactam antibiotics as well as penicillinase-resistant penicillins like methicillin and oxacillin. Recently, MRSA was found to be resistant not only to β-lactam antibiotics but also multidrug-resistant (MDR) [1, 2]. MRSA infection consequently has a high rate of morbidity and mortality.

*Staphylococcus aureus* typically colonizes the skin and mucous membranes of asymptomatic, possibly healthy people and animals. The colonization of MRSA in animals has received intensive attention since animals may play a crucial role in the reservoir of human infection. Numerous reports discuss the potential for MRSA transmission between humans and animals through close contact is mentioned in many reports [3–8]. Even though it is unclear how often MRSA spreads from animals to people, it still poses a serious threat to medical and veterinary research. Methicillin-resistant *Staphylococcus aureu*s has been found in healthy animals, including dogs, cats, horses, donkeys, sheep, goats, cattle, and swine, according to several reports [3, 8–18]. However, the investigation of MRSA in rabbits is less information, especially in healthy rabbits.

This study aimed to estimate the prevalence of MRSA in healthy rabbits, the antimicrobial resistance profile of MRSA isolates, and the antimicrobial-resistant genes that may be hidden in MRSA isolates.

## Materials and Methods

### Ethical approval

Sample collection was performed by an expert veterinarian and carried out in accordance with Guide for the care and use of laboratory animals. All experimental protocols involving animals were approved by The Faculty of Veterinary Science-Animal Care and Use Committee, Mahidol University (protocol number MUVS-2014-42).

### Study period and location

The study was conducted from January 2015 to March 2016. The samples were collected at Prasu-Arthorn Animal Hospital, Faculty of Veterinary Science, Mahidol University, Nakhonpathom Province.

### Specimen collection and bacterial isolation

The samples were taken from 44 healthy rabbits that went to the Prasu-Arthorn Animal Hospital in the Thai province of Nakornpathom, 56 km South of Bangkok, for vaccinations and health checks. Specimens collections were consent from the owners. Forty-two eye swabs, 44 ear swabs, 44 oral cavity swabs, 44 ventral thoracic area skin swabs, and 44 perineal area swabs were among the 218 specimens. All samples were transported in an Oxoid transport medium (UK) and delivered within 8 h to the Veterinary Science Department’s Microbiological lab at Mahidol University. Mannitol salt agar (MSA, Oxoid, UK) was applied to each specimen, and it was then incubated at 37°C for 24–48 h. As a result of mannitol fermentation, three suspected yellow colonies on the MSA of each specimen were selected and subcultured into the blood agar (Oxoid, UK). The isolated colonies on blood agar were seen to exhibit a hemolysis pattern, and they were further identified using traditional biochemical techniques such as Gram staining (Merck, Germany), catalase production (Merck, Germany), coagulation (Ramel, Oxoid, UK), and commercial latex agglutination to detect protein A (Dryspot starchitect plus, Oxoid, UK).

### Antimicrobial susceptibility test

According to the Clinical and Laboratory Standards Institute (CLSI) guideline, antimicrobial susceptibility testing and interpretation were carried out using the Kirby–Bauer disk diffusion method [19]. A total number of 13 antimicrobial drugs were tested: Amikacin (30 μg), azithromycin (15 μg), cefazolin (30 μg), cefoxitin (30 μg), ceftriaxone (30 μg), chloramphenicol (30 μg), ciprofloxacin (5 μg), doxycycline (30 μg), gentamicin (10 μg), moxifloxacin (5 μg), norfloxacin (10 μg), penicillin (10 units), and trimethoprim/sulfamethoxazole (1.25 μg/23.75 μg). As a standard strain, *S. aureus* ATTC^®^ 25923 was used. The inhibition zone was measured and identified as susceptible (S), intermediate (I), and resistant (R) following the CLSI guidelines [19]. According to the recommendations, MRSA was distinguished by a phenotype that was cefoxitin-resistant.

### Detection of antimicrobial resistance genes

Bacterial genomic DNA was extracted by DNA extract kit (Geneaid, Taiwan). In a nutshell, the lysis buffer was added to lyse the bacterial cells. In the extraction column, DNA was attached to the membrane and was lysed by adding the lysis buffer. DNA was bound to the membrane in the extraction column and was eluted by an elution buffer. Using a Nanodrop one (Thermo-Scientific, Germany), the absorbance at 260 nm (A260) was measured to calculate the amount of DNA that had been extracted. Drug-resistant genes (*bla*Z, *mec*A, *aac*A*-aph*D, *msr*A, *tet*K, *gyr*A, *grl*A, and *dfr*G) were amplified using a specific primer pair and polymerase chain reaction (PCR) using the extracted DNA as a template. The nucleotide sequences of specific primers are listed in Table-1 [20–24]. One mM of each primer, 2.5 mL of 10 U Taq PCR buffer, 0.2 mM dNTP, 2 mM MgCl_2_, 1 U of Taq DNA polymerase (Thermo-scientific, Germany), and 1 ug of DNA template made up the 25 mL total volume of the PCR mixture. The PCR mixture was subjected to amplify the target gene with the following thermal cycle conditions using the Flexcycler^2^ (Analytik Jena, Germany): 30 cycles of amplification at 95°C for 30 s, annealing temperature for 30 s for each primer, 72°C for 60 s, and final extension at 72°C for 10 min was performed after 5 min at 95°C. The positive control strains for detecting antimicrobial-resistant genes were obtained from the previous study [25]. SYBR-safe (Invitrogen, USA) staining and 1.5% agarose gel electrophoresis were used to analyze the amplicons. The DNA bands were observed under the ultraviolet transilluminator (UVP Bio-imaging system, Invitrogen, USA).

**Table-1:** The PCR primer for the amplification of antimicrobial resistance genes.

Target genes	Primer (5′–3′)	PCR amplicon (bp)	Reference
Beta-lactams			
*bla*Z	CAGTTCACATGCCAAAGAG TACACTCTTGGCGGTTTC	772	[20]
*mec*A	AAAATCGATGGTAAAGGTTGGC AGTTCTGCAGTACCGGATTTGC	533	[21]
Aminoglycosides			
*aac*A-*aph*D	CAAGAGCAATAAGGGCATAC CAATAGTTTCAATAGGATAA	936	[22]
Quinolone			
*gyr*A	AATGAACAAGGTATGACACC TACGCGCTTCAGTATAACGC	223	[23]
*grl*A	ACTTGAAGATGTTTTAGGTGAT TTAGGAAATCTTGATGGCAA	459	[23]
Macrolides			
*msr*A	GGCACAATAAGAGTGTTTAAAGG AAGTTATATCATGAATAGATTGTCCTGTT	940	[20]
Tetracyclines			
*tet*K	TTATGGTGGTTGTAGCTAGAAA AAAGGGTTAGAAACTCTTGAAA	348	[21]
Trimethoprim			
*dfr*G	CCCAAGGACTGGGAATATG TCCTCATAATTCACTTCTGG	326	[24]

PCR=Polymerase chain reaction

### Statistical analysis

All data were organized and analyzed using GraphPad Prism version 9 (La Jolla, CA, USA). Antimicrobial resistance phenotypes of MRSA isolates and the prevalence of MRSA in each position are expressed as percentages.

## Results

**Figure-1:**
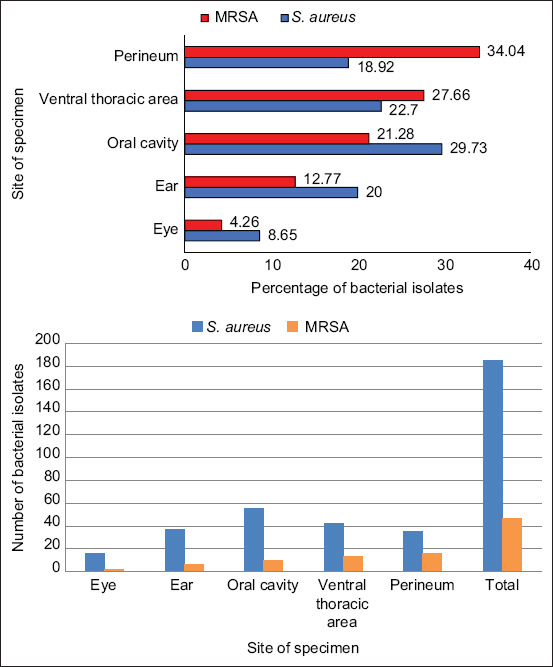
Methicillin-resistant *Staphylococcus aureus* and *S. aureus* isolated from each position of healthy rabbits.

**Figure-2:**
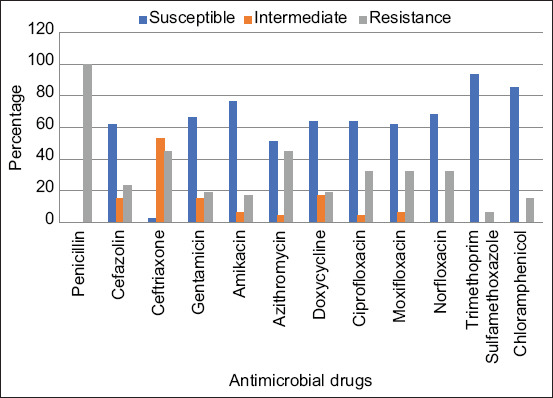
Antimicrobial susceptibility test of methicillin-resistant *Staphylococcus aureus* isolated from healthy rabbits.

From 218 samples taken from 44 healthy rabbits, 185 *S. aureus* were isolated, with the majority of these samples coming from the oral cavity (29.73%) and ventral thoracic area (22.7%). Forty-seven (25.41%) MRSAs were found in *S. aureus* isolates, with the majority of these being found in the perineum (34.04%) and ventral thoracic area (27.66%) of the specimen (Figure-1). Among MRSAs, 61.7% of isolates were MDR strains. The isolated MRSAs were analyzed for antimicrobial susceptibility testing, as shown in Figure-2. In this study, penicillin-resistant bacteria were primarily isolated (100%) and ceftriaxone (44.68%) and azithromycin (44.68%) were the next most common antibiotics. Penicillin, cefazolin, ceftriaxone, gentamicin, azithromycin, ciprofloxacin, moxifloxacin, norfloxacin, and chloramphenicol were among the nine antimicrobial drugs with which MRSA had a high level of resistance (61.7%). Table-2 shows the antimicrobial resistance pattern of MRSA.

**Table-2:** Antimicrobial susceptibility pattern and antimicrobial resistance genes of MRSA isolated from healthy rabbits.

Sample no	Sample code	Source	Antimicrobial susceptibility pattern	Antimicrobial-resistant gene
1	N1S1	Ventral thoracic area	P, KZ, CRO, CN, CIP, MXF, NOR, AZM, C	*bla*Z
2	N1S2	Ventral thoracic area	P, KZ, CRO, CN, CIP, MXF, NOR, AZM, C	*bla*Z
3	N1S3	Ventral thoracic area	P, KZ, CRO, CN, CIP, MXF, NOR, AZM, C	*bla*Z
4	N2E2	Ear	P	
5	N2E3	Ear	P, KZ, AK	
6	N2S1	Ventral thoracic area	P, DO	*bla*Z*, tet*K
7	N2S2	Ventral thoracic area	P, KZ, CRO, CN, CIP, NOR, AZM	*bla*Z*, msr*A*, gyr*A*, grl*A
8	N3O1	Oral cavity	P, CRO, CN, CIP, MXF, NOR, DO, SXF	*bla*Z*, aac*A*-aph*D*, tet*K*, dfr*G*, gyr*A*, grl*A
9	N3S1	Ventral thoracic area	P, AZM	
10	N3S2	Ventral thoracic area	P, AZM	
11	N3S3	Ventral thoracic area	P, KZ, CIP, MXF, NOR, SXF, C	*gyr*A
12	N8Ey1	Eye	P, CN, CIP, MXF, NOR, AZM, DO, C	*aac*A*-aph*D*, tet*K*, gyr*A*, grl*A
13	N10P2	Perineum	P, CIP, MXF, NOR, AZM, DO, SXF, C	*bla*Z*, dfr*G*, gyr*A
14	N15O1	Oral cavity	P, CRO, CIP, MXF, NOR, AZM,	*bla*Z*, gyr*A*, grl*A
15	N16P1	Perineum	P, KZ, AK,	
16	N16P3	Perineum	P, KZ, CRO, AK, MXF	*gyr*A
17	N20S1	Ventral thoracic area	P, CRO	
18	N20S2	Ventral thoracic area	P	
19	N22Ey2	Eye	P	
20	N23O2	Oral cavity	P	
21	N24P1	Perineum	P, CRO, AK	
22	N24P3	Perineum	P, KZ, CRO, AK	
23	N26S3	Ventral thoracic area	P, AK	*bla*Z
24	N27P2	Perineum	P, CRO, AK	
25	N27P3	Perineum	P, CRO, AK	
26	N28E3	Ear	P, CRO, CIP, MXF, NOR, C	*gyr*A
27	N30O1	Oral cavity	P	*bla*Z
28	N30O2	Oral cavity	P	*bla*Z
29	N30O3	Oral cavity	P	*bla*Z
30	N35P2	Perineum	P	
31	N36S1	Ventral thoracic area	P, CN, CIP, MOX, NOR, AZM	*bla*Z*, aac*A*-aphD, msr*A*, gyr*A*, grl*A
32	N36S3	Ventral thoracic area	P, CRO, CIP, MOX, NOR, AZM	*msr*A*, gyr*A*, grl*A
33	N37E2	Ear	P, CRO, CIP, MOX, NOR, AZM	*bla*Z*, gyr*A*, grl*A
34	N40O2	Oral cavity	P, CRO, AZM	
35	N40O3	Oral cavity	P, CRO, AZM	
36	N42O3	Oral cavity	P	
37	N42E2	Ear	P	*bla*Z
38	N42P2	Perineum	P, CN, CIP, MOX, NOR, AZM	*bla*Z, *aac*A-*aph*D, *gyr*A
39	N42P3	Perineum	P, CN, CIP, MOX, NOR, AZM	*bla*Z, *aac*A-*aph*D, *gyr*A
40	N43E3	Ear	P, AZM, DO	*bla*Z, *tet*K
41	N43P1	Perineum	P, CRO	
42	N43P2	Perineum	P, CRO	
43	N43P3	Perineum	P, CRO	
44	N44O1	Oral cavity	P, AZM, DO	*bla*Z, *tet*K
45	N44P1	Perineum	P, CRO, AZM, DO	*bla*Z, *tet*K
46	N44P2	Perineum	P, CRO, AZM, DO	*bla*Z, *tet*K
47	N44P3	Perineum	P, CRO, AZM, DO	*bla*Z, *tet*K

MRSA=Methicillin-resistant *Staphylococcus aureus*, AK=Amikacin, AZM=Azithromycin, C=Chloramphenicol, CIP=Ciprofloxacin, CN=Gentamicin, CRO=Ceftriaxone, DO=Doxycycline, KZ=Cefazolin, MXF=Moxifloxacin, NOR=Norfloxacin, P*=*Penicillin, SXF=Trimethoprim/Sulfamethoxazole

Antibiotic resistance genes were identified in the isolated MRSAs. G*yr*A carried 36.17%, *tet*K 23.4%, and *bla*Z 47.83% of MRSA, respectively. Moreover, some isolate carried more than one antimicrobial-resistant gene, for example, a combination of *bla*Z, *aac*A*-aph*D, *tet*K, *dfr*G, *gyr*A, and *grl*A. Genes that are resistant to antibiotics are listed in Table-2.

## Discussion

Among *S. aureus*, MRSA is a critical concern since they usually have MDR capacity. Unfortunately, MRSA can be found in healthy people and animals as well as the infected ones. Methicillin-resistant *Staphylococcus aureus* has been found in healthy animals such as dogs [8, 10, 13], cats [8, 9], horses [15], donkeys [12], sheep [11], goats [16, 17], cattle [16], and swine [18], according to several reports. However, there have been relatively few studies on MRSA isolated from rabbits. In addition, the prevalence of MRSA in healthy farm animals is quite high compared with companion animals. Determining the prevalence and antimicrobial resistance profile of MRSA that was isolated from healthy rabbits was the goal of this study.

*Staphylococcus aureus* can localize on various body parts in rabbits, including the ventral thoracic region, skin, ears, and oral cavity. Chai *et al*. [4] reported that the ears and the oral cavity were common sites where *S. aureus* could be found. The oral cavity specimens (29.73%) and ventral thoracic area skin specimens (22.7%) contained the greatest number of *S. aureus* isolates in this study. Methicillin-resistant *Staphylococcus aureus* made up 25.41% of the isolated *S. aureus* and was most frequently found in skin samples from the perineal area (34.04%) and ventral thoracic area (27.66%).

Methicillin-resistant *Staphylococcus aureus* is usually resistant to several antimicrobial drugs and preserves numerous resistant genes. In contrast to other animals, rabbits typically only receive a small number of antimicrobial medications. The antimicrobial susceptibility pattern of *S. aureus* isolated from healthy rabbits was also examined in this study. The multidrug resistance of MRSA was confirmed in this study (61.7%). Multidrug-resistant MRSA had all of the antimicrobial-resistant genes that we examined. The most frequent gene discovered in this study was *bla*Z. Resistance to penicillin is consistent with this. We found that many bacterial isolates carried *gyr*A and indicated possible quinolone resistance. Fewer isolates that were azithromycin-resistant also carried the *mrs*A gene. This may suggest that these macrolide-resistant isolates of azithromycin have additional defense mechanisms, such as the production of encoded methyltransferases by the *erm* gene and macrolide efflux pumps mediated by the *mef* gene, which have been described by Fyfe *et al*. [26]. The antimicrobial resistance in healthy rabbits should be monitored since rabbits as pets are increasingly becoming popular.

The previous research found MRSA in a variety of animal-related environmental samples, dust, farmed rabbits, and wipes [27]. According to Friese *et al*. [27], MRSA was found in turkey and broiler barns as well as on nearby soil and airborne surfaces. The samples were taken from animals, the environment they were in (including the air), the barns’ surroundings (including ambient air and boot swabs of ground surfaces at various distances from the barn), and the animals themselves. They found that MRSA was detected in the air of most barns, as well as in many samples originating from animals. As a result, it seems possible that MRSA could spread within poultry farms as well as through the air. In addition, it was discovered that animal handlers who had frequent contact with both pets and animals were more likely to have MRSA in their bodies than the animals they were caring for [28]. Thus, healthy rabbits in this study possibly derived MRSA from their environment as well as from their owner.

The transmission of pathogenic bacteria between humans and animals is an increasing concern, especially MDR microorganisms. Multidrug-resistant microbial infections complicate medical management, lengthen hospital stays, and cost money. One of the most significant opportunistic bacteria is *S. aureus*. It colonizes the many sites of the body both in humans and animals, without any symptoms. However, it can lead to serious issues such as sepsis, endocarditis, and osteomyelitis.

## Conclusion

The owner’s health is at risk because MRSA is a significant human pathogen and is found in rabbits. In contrast, healthy rabbits possibly get these bacteria from their owners or environment. However, these results point to the existence of MRSA that is resistant to antibiotics in healthy rabbits. Therefore, it is necessary to control rabbit antibiotic use to stop the spread of antibiotic resistance. Antimicrobial stewardship programs need to be conducted to educate rabbit owners. The degree of virulence displayed by these bacteria should be evaluated to assess potential pathogenesis.

## Authors’ Contributions

SB and NI: Conceived and designed the experiments. NS: Performed sample collection and animal care. AJ, SS, TK, and WT: Performed the experiments. AJ, NS, NI and SB: Analyzed the data. SB: Contributed reagents, materials, and analysis tools. AJ, SB, and NI: Wrote and edited the manuscript. All authors have read and approved the final manuscript.
